# The Application of Deep Learning in Cancer Prognosis Prediction

**DOI:** 10.3390/cancers12030603

**Published:** 2020-03-05

**Authors:** Wan Zhu, Longxiang Xie, Jianye Han, Xiangqian Guo

**Affiliations:** 1 Department of Preventive Medicine, Institute of Biomedical Informatics, Cell Signal Transduction Laboratory, Bioinformatics center, School of Basic Medical Sciences, Henan University, Kaifeng 475004, China; xielongxiang123@126.com; 2 Department of Anesthesia, Stanford University, 300 Pasteur Drive, Stanford, CA 94305, USA; 3 Department of Computer Science, University of Illinois, Urbana Champions, IL 61820, USA; mr.neohan@gmail.com

**Keywords:** cancer prognosis, deep learning, machine learning, multi-omics, prognosis prediction

## Abstract

Deep learning has been applied to many areas in health care, including imaging diagnosis, digital pathology, prediction of hospital admission, drug design, classification of cancer and stromal cells, doctor assistance, etc. Cancer prognosis is to estimate the fate of cancer, probabilities of cancer recurrence and progression, and to provide survival estimation to the patients. The accuracy of cancer prognosis prediction will greatly benefit clinical management of cancer patients. The improvement of biomedical translational research and the application of advanced statistical analysis and machine learning methods are the driving forces to improve cancer prognosis prediction. Recent years, there is a significant increase of computational power and rapid advancement in the technology of artificial intelligence, particularly in deep learning. In addition, the cost reduction in large scale next-generation sequencing, and the availability of such data through open source databases (e.g., TCGA and GEO databases) offer us opportunities to possibly build more powerful and accurate models to predict cancer prognosis more accurately. In this review, we reviewed the most recent published works that used deep learning to build models for cancer prognosis prediction. Deep learning has been suggested to be a more generic model, requires less data engineering, and achieves more accurate prediction when working with large amounts of data. The application of deep learning in cancer prognosis has been shown to be equivalent or better than current approaches, such as Cox-PH. With the burst of multi-omics data, including genomics data, transcriptomics data and clinical information in cancer studies, we believe that deep learning would potentially improve cancer prognosis.

## 1. Current Development in Cancer Prognosis Prediction

In the United States, approximately 1 in 10 adults have been diagnosed with cancer [1]. Cancer causes 1 in 6 deaths around the world [1]. While new therapies can improve cancer treatment and increase survival rate, cancer prognosis is to estimate cancer development, to provide survival estimation and to improve clinical management. One major task in cancer prognosis is to provide better survival estimation based on patients’ clinical features and molecular profile. 

Current state-of-the art analytic methods in cancer prognosis for survival analysis are statistical approaches, including Cox proportional hazard regression [2,3], Kaplan Meier estimator [4] and log-ranks test [5,6,7]. The main data sources for these approaches in cancer prognosis for survival prediction are mainly clinical data, including cancer diagnosis, cancer types, tumor grades, molecular profile, etc. In recent years, more data types are available to better understand disease status. These data are high throughput and high dimensional multi-omics data of patient samples [8]. Multi-omics data include genomic data (i.e., whole genome data, single nucleotide polymorphism (SNP) data, copy number alternation (CNA) data, etc.), expression data (i.e., mRNA and miRNA data), proteomic data and epigenetic data (i.e., methylation and other chromosomal modifications). The volume of multi-omic data has poses challenges to use purely statistical methods to perform prediction. Other methods, including machine learning approaches, have been applied or established to solve these problems. So far, some machine learning methods, including principal component analysis (PCA), clustering and autoencoder, have been tested to classify cancer types [9,10]. Also, machine learning methods, including support vector machine (SVM), Bayesian network, semi-supervised learning and decision tree, have been applied to cancer prognosis prediction and shown some good success [11,12,13,14,15,16]. 

The establishment of public accessible large-scale cancer databases indeed provides an open-source platform for researchers and clinicians to share and analyze patients’ multi-omics data. The Cancer Genome Atlas (TCGA), Gene Expression Omnibus (GEO) and Genotype-Tissue Expression (GTEx) databases are the major ones. The Cancer Genome Atlas (TCGA) database has clinical and molecular data of over 11,000 tumor patients across 33 different tumor types [17,18], including genomic (whole genome and/or exome sequencing, WGS/WES), transcriptomic (RNAseq, small RNAseq), epigenomic (HumanMethylation450 BeadChip) and proteomic profiling (reverse-phase protein arrays, RPPAs) data. There are several public portals of TCGA, such as TCGA data portal [19], cBioPortal [20], the University of California, Santa Cruz cancer genome browser (UCSC Xena) [21] and FIREHOSE [22]. The Gene Expression Omnibus (GEO) database is a public data repository storing microarray and next-generation sequencing (NGS) data, as well as other high-throughput functional genomic datasets, such as genome methylation, chromatin structure, genomic mutation/copy number variation, protein profiling, and genome–protein interactions [23,24]. The Genotype-Tissue Expression (GTEx) database contains whole-genome sequencing and RNA-sequencing profiles from ~960 postmortem adult donors of many tissue samples that have tissue images stored in an image library for public access [25,26]. These public data not only provide unprecedented opportunities to better illustrate the molecular mechanism of cancers and normal tissues, but also become the major resources to apply novel methods, build models and perform predictions in cancer prognosis.

## 2. Overview of Deep Learning

Deep learning, also known as deep neural network (DNN), is a branch of machine learning that has made some major breakthrough in recent years due to the increase of computation power, the improvement in model architecture [27] and the exponential growth of data captured by cellular and other devices. There are three basic machine learning paradigms, supervised learning, unsupervised learning and reinforcement learning. Supervised learning algorithm are those should be fed in a set of training data containing features (inputs) and labels (outputs). Some popular supervised learning algorithms include linear and logistic regression [28], SVM [29], naive bayes [30], gradient boosting [31,32], classification trees, and random forest [33,34]. These methods are commonly used in classification and regression studies. Unsupervised learning, on the other hand, does not require pre-existing output/labels and aim to find patterns based on the input data distributions. Clustering (e.g., hierarchical clustering [35,36], K-means [37,38]) is the most common unsupervised learning method. Latent Dirichlet Allocation (LDA) [39], PCA [40] and word2vec [41], are among the most recent popular unsupervised learning approaches. Neural network (NN) can either be supervised, unsupervised or semi-supervised learning, suggesting its flexibility. Reinforcement learning [42] can be summarized as a reward system for the computer program to maximize the rewards in order to search for the best solution [27].

Deep learning (or DNN) consists of multiple layers of artificial neurons that mimic neurons in human brain. Similar to linear regression, each neuron has a weight value that is updated by gradient descent algorithm during backpropagation to minimize global loss function [43]. By applying nonlinearity using activation function, such as sigmoid, tanh, or relu, to the multiple layers of each neuron, more abstract mathematical relationship was extracted from the input data to map to the output [44]. A well trained model can therefore be used to predict new unlabeled data. Deep learning is a branch of machine learning, and therefore inherits some common knowledge foundation in machine learning, including basic probability and statistics, loss/cost function and etc., but in the meantime has more flexibility and can be built towards more complex layers and multiple neurons in each layer to have better predictive power [45,46,47,48,49,50]. The most commonly used NN in medical research includes fully connected NN (or simplified as NN) for structured data, convolutional NN (CNN) for image data, and recurrent NN (RNN) for text and sequence data. 

In recent years, deep learning has been applied to biomedical research to annotate pathogenicity of genetic variants [51,52], show state-of-the-art performance in the task of genomic variant calling [53] and improve protein folding prediction [54,55]. Compared to other methods, deep learning is more flexible and generic to be applied on discrete or continuous data [56], requires less feature engineering with expertise knowledge compared to machine learning in general [27] and works better than many state-of-the-art methods [53].

## 3. Current Application of Deep Learning in Cancer Prognosis 

To review the application of deep learning in the field of cancer prognosis, we used key words, including “deep learning”. “neural networks” and “cancer prognosis”, and searched literature on PubMed. To better understand the development of the field and for better comparison, we have included studies that built simple NN models which consist of 3–4 layers and studies that built DNNs which consist of more than 4 layers. We reviewed and summarized these studies and models. Based on the types of NN and whether feature extraction has been used, the publications that we reviewed could be grouped into three classes: (1) NN models with no feature extraction, (2) Feature extraction from multi-omics data to build fully connected NNs, and (3) CNN based models. Here, we reviewed and summarized these studies and models.

### 3.1. NN Models with no Feature Extraction

As mentioned, Cox proportional hazards model (Cox-PH) is a multivariate semi parametric regression model that has been used widely in cancer studies to compare survival characteristics between two or more treatment groups [2,57]. Some early attempts in cancer prognosis have either used clinical tumor and patient data [58], cellular features from tissue slides [14] or some genes expression data [13] to build the models. To show the performance, these studies compared the performance of NN to Cox-PH and/or Kaplan Meier methods, and showed that simple NN models have achieved similar performance compared to these methods (Table 1). Also, in these studies, because the number of features was relatively small without omics data, feature selection was not necessary.

Since the wide acceptance of Cox regression model in survival prediction, Cox regression was used as the output layer to build NNs to predict cancer survival. Cox-nnet [59] is a NN network which used genomic data from TCGA as input and Cox regression as the output layer. To avoid overfitting, they tested ridge regularization, dropout, reduction of NN complexity by using 0 to 2 hidden layers and a combination of ridge and dropout in training the NN (Table 1). They reported that dropout and reduction of NN complexity by using 1 hidden layer worked the best to avoid overfitting in their experimental setting. To measure the performance, they showed that Cox-net performed better than Cox-PH, Cox-boost (based on gradient boosting) or random forest in the TCGA datasets that they have tested (Table 1). 

Katzman et al. has built a neural network model, named DeepSurv, to perform survival analysis. DeepSurv is a feed forward NN that uses patient’s clinical data as input and applied dropout, learning rate decay, regularization, and other commonly used hyperparameters to optimize for different datasets [60]. Their results showed that this model performed better than CoxPH models (Table 1). Another neural network model, named RankDeepSurvival, has adapted the basic architecture of DeepSurv and increased the depth of the network to build 3–4 hidden layers’ DNN to perform survival analysis in multiple datasets, including cancer datasets [61]. More importantly, they have updated the loss function by using the sum of mean squared error loss and a pairwise ranking loss based on ranking information on survival data [61]. They reported that RankDeepSurivival model outperformed CoxPH models and DeepSurv model in breast cancer datasets from Molecular Taxonomy of Breast Cancer International Consortium (METABRIC) and the German Breast Cancer Study Group (GBSG) (Table 1). Both of these studies have further validated their models performed better than CoxPH models in other disease datasets, such as heart disease and diabetes, which suggested that deep learning models can be generalized for different tasks. 

### 3.2. Feature Extraction from Gene Expression Data to Build Fully Connected NNs

Health data has the characteristics of high-dimension, small sample size and complex non-linear effects between biological components [62,63]. Dimension reduction assists the integrative analysis of multi-omics data [64]. These following studies have tested different algorithms to reduce dimension of sequencing data, extract a smaller number of features and train a fully connected NN.

In a study to predict breast cancer prognosis, Sun et al. used a method named minimum redundancy maximum relevance (mRMR) [65] to reduce the dimensionality of gene expression data and copy number alternation (CNA) data by extracting 400 and 200 genes, respectively, from these datasets [66]. Next, 3 NN models were built using features selected from gene expression data, CNA data or clinical data, respectively. Finally, prediction outputs of these three NN models were added up based on a weighted linear aggregation to calculate a final prediction score. They named this model as Multimodal Deep Neural Network by integrating Multi-dimensional Data (MDNNMD). When they selected threshold of 0.443–0.591, a high specificity (0.95–0.99), yet low sensitivity (0.2–0.45) were reported (Table 2). To show model performance, they reported that ROC (0.845), accuracy, and precision, and Matthew’s correlation coefficient (MCC) of MDNNMD outperformed other methods, including SVM, random forest, and logistic regression (Table 2). One of the reasons that the model has a big performance difference between specificity and sensitivity is likely due to the imbalanced data in training the NN (491 short term survival versus 1489 long term survival cases). 

There are many ways to reduce data dimensionality. Huang et al. have obtained five omics data, including gene expression (mRNA) data, miRNA data, copy number burden data, tumor mutation burden data and clinical data, performed feature extraction from these data and built a deep learning model to predict breast cancer patient survival [67]. They also applied a Cox proportional hazards model to develop a survival analysis learning with a multi-omics NN (or SALMON) model [67]. In this model, input layers were comprised of features extracted from mRNA and miRNA data using a local maximal Quasi-Clique Merger (lmQCM) algorithm inspired by spectral clustering [70]. A matrix, named eigengene, was generated from lmQCM algorithm and used to represent 57 and 12 dimensions from mRNA and miRNA data, respectively (Table 2). In the hidden layer, mRNA and miRNA data comprises 8 and 4 neurons, respectively. Adam optimizer and lasso regularization were used as hyperparameters in training (Table 2). Sigmoid function was used as activation function after each forward propagation to introduce non-linearity and Cox proportional hazards regression and was used as the output to predict survival time. This model achieved a median concordance index (c-index) [71] of 0.728 which has been suggested to outperform other models that didn’t include high dimensional features extracted from mRNA and miRNA data (Table 2), suggesting feature extraction improves model performance.

In addition to reducing data dimension using algorithm, feature extraction by application of domain knowledge as selection criteria has also been tested. Hao et al. used gene expression data from 475 glioblastoma multiforme patients with ~12 k genes that contained survival information to build a prognosis model [62] (Table 2). They grouped the samples into two groups, long term survival (LTS, survival time >= 24 months) and non-long term survival (non-LTS, survival time <24 months). Next, they used pathway data from the Molecular Signatures Database (MSigDB) and mapped 4359 genes to 574 pathways. They constructed a NN using the 4,359 genes as input and 574 pathways as the first hidden layer and applied dropout and L2 regularization to avoid overfitting. Since 20% of the samples are LTS, the training data suffered from imbalanced data. It is a common problem in handling patient data. They suggested PASNet achieved AUC of 0.66 that is better than the performance of logistic LASSO, random LASSO or SVM model. The advantage of PASNet is that it took biological pathways into consideration when building a NN model.

NN itself can be used to extract features from multi-omics data. Hepatocellular carcinoma (HCC) is the most common type of liver cancer. High heterogeneity of the disease makes the prognosis prediction challenging. Chaudhary et al. built a NN model using multi-omics data of 360 HCC samples from TCGA database [68]. The multi-omics data includes mRNA expression, miRNA expression, CpG methylation and clinical data. They used unsupervised autoencoder NN to transform features and perform dimension reduction [68] and extract 100 feature nodes from miRNA, mRNA and methylation data (Table 2). Next, they used a Cox-PH model to identify 37 significant features, applied K-means clustering to identify survival risk and used ANOVA to get feature ranking. Finally, prognosis prediction was built using a SVM model. In another study, Shimizu et al. picked 23 genes from 184 prognosis related genes based on the statistical significance of these individual genes on the overall survival of breast cancer patients [69]. They used gene expression levels of these 23 genes to build a NN to get gene weights from NN’s nodes and generate a molecular prognostic score (mPS) (Table 2). The mPS was then applied to evaluate prognosis. Although both studies didn’t report the performance of the NNs in their study, these studies suggested that NN can also be a useful tool for dimension reduction of multi-omic data for prognosis prediction.

### 3.3. CNN-Based Models 

In recent years, deep learning approach has been made the most significant progress because state-of-the-art networks have been built using convolutional NN (CNN) [45,46,47,48] and recurrent NN (RNN) [49,50]. Many success have been shown in the areas of image recognition/classification and computer vision by CNN, and natural language processing (NLP) and sequencing data investigation by RNN. Specifically, great performance has also been witnessed in many medical areas, including classification of skin cancer types [72,73], identification of pathological histological slides [74], identification of Aβ plague region in Alzheimer’s patients, classification of cancer cells from normal cells using nuclear morphometric measure [75], and extraction information from electronic health records (EHR) to predict hospital readmission [76,77], mortality [78], and clinical outcome [79]. In cancer prognosis studies, CNN has been applied to the classification of cancerous tissue for survival prediction or extraction of feature for downstream prognosis. Some of these studies also added RNN layers to extract sequential information from the data.

Glioblastoma multiforme (GBM) is a type of brain tumor. Methylation of O6-methylguanine methyltransferase (MGMT) gene promoter has been found to associate with longer survival and better response to a drug, temozolomide. Therefore, methylation of MGMT gene has been considered as a biomarker. However, verification of MGMT gene promoter in the brain is difficult and invasive. Using high quality MRI images from patients that have labeled information of methylation status of MGMT promoter, a 50-layer pre-trained CNN model, ResNet50 [80] was used for transfer learning and achieved the highest accuracy of ~95% compared to ResNet18 and ResNet34 [81] (Table 3). Similarly, another research group used brain MRI images from a different cohort of GBM patients to build a bidirectional convolutional recurrent NN (CRNN) model to predict methylation status of MGMT gene promoter and suggested patient’s sensitivity to temozolomide based on the prediction of methylation status [82]. RNN layers were added into this model to capture sequential information of MRI images [82], but the effect was not well studied since the model performance was not compared with or without RNN layer. In this study, the authors applied many techniques to reduce overfitting, such as L2 regularization, dropouts, and data augmentation (Table 3). Although the training accuracy is high (0.97), but validation and test accuracies were only 0.67 and 0.62, respectively, suggesting the model was still overfit to the training data. Instead of predicting methylation status of MGMT gene promoter in glioblastoma cancer, Mobadersany et al. trained a survival convolutional NN (SCNN) using histology images, clinical data with or without genomic markers in glioma and glioblastoma and showed the prediction power of this NN has surpassed the prognostic accuracy of the WHO genomic classification and histologic grading in 2018 [83]. Using H&E-stained tissue sections of 1,061 samples from 769 patients, regions of interest (ROIs) that contain viable tumor cells by a web-based platform were identified in tissue images to train a CNN with Cox proportional hazard regression as the output layer to predict patient outcomes (Table 3). They also compared the performance of the NN with or without inclusion of some genomic data (i.e., IDH gene mutation and 1p/19q codeletion). They showed that with the addition of genomic data, the performance has improved the median of c-index from 0.754 to 0.801 (Table 3).

Colorectal cancer (CRC) is a type of solid tumors. H&E images are the major tool to diagnose CRC and determine the stage of CRC. In H&E slide of CRC patients, it is important to differentiate normal tissues from cancer regions. Kather et al. [74] hand labeled 100,000 image patches using 86 CRC H&E slides into 9 tissue classes, including adipose, background, debris, lymphocytes, mucus, smooth muscle, normal mucosa, stroma and cancer epithelium [74]. They used these images as the training data with an additional of 7,180 images from 25 patients as the testing data to build a CNN model using state-of-the-art CNN networks, such as VGG19 and Resnet50, to perform transfer learning and have reached 94–99% accuracy in classifying tissue types (Table 3). By calculating the hazard ratios (HRs) for shorter overall survival (OS) and selecting optimal cutoffs based on the ROC curve, the authors defined a deep stromal score and suggested although not significant correlated, deep stromal score shows a trend of correlation to shorter OS. In another CRC study, Bychkov et al. [84] used CNN models as a tool for feature extraction and built an RNN (LSTM) model to predict CRC patient survival. They used VGG16 as the base model to perform transfer learning and extracted a 256-tile feature vector from each input H&E image of tumor tissue microarray (Table 3). They then input these feature vectors of 220 patients (equal number of patients in short and long term survival group) to train a LSTM-cell RNN model. They also trained SVM, naive bayes and logistic regression models to compare the performance. They showed that LSTM model reached an AUC of 0.69, while SVM, naive bayes, and logistic regression reached AUCs of 0.64, 0.61, 0.65, respectively. They also reported that human experts can only reach an AUC of 0.57–0.58, suggesting that the performance of this model is better than human.

Malignant mesothelioma is a type of rare and highly lethal cancer of the pleural lining. According to the WHO classification, patients tissue biopsy can be classified into epithelioid, sarcomatoid and biphasic types. Prognosis of mesothelioma is closely associated with tissue types as epithelioid type has the longest overall survival, sarcomatoid type has the shortest overall survival and biphasic type is in-between [85]. Based on the clinical knowledge, Courtiol et al. built a MesoNet model using 100 to 10,000 tiles of histological tissue from 2,300 H&E slide from the MESOPATH/MESOBANK database. By transfer learning of ResNet50 and performing feature extraction, a matrix of features (2,048) was extracted from each tile to train MesoNet. C-index showed that MesoNet performed better than histological based classification methods, but not as good as a linear regression based model, named Meanpool (Table 3) [85].

Similarly, CNN models can be used to extract features from images for building other machine learning model to predict cancer prognosis. High-grade serous ovarian cancer (HGSOC) is the most common and most lethal histological type of ovarian cancer. Wang et al. [86] used CT-based images and trained a CNN model to extract image features for building a Cox-PH survival prediction model. In this study, 102 HGSOC patients, who underwent debulking surgery and have remained in 2-year follow-up study, were used as a feature extraction cohort (Table 3). A total of 8,917 tumor images were used to train an unsupervised CNN model for feature extraction of a 16-dimensional feature vector. Next the feature vector was fed to a multivariate Cox-PH regression model to identify the association of feature vector and recurrence of HGSOC. This study provides an example of using NN, particularly CNN, to extract image features for downstream studies.

## 4. Challenges in the Application of Deep Learning in Cancer Prognosis

**Figure 1:**
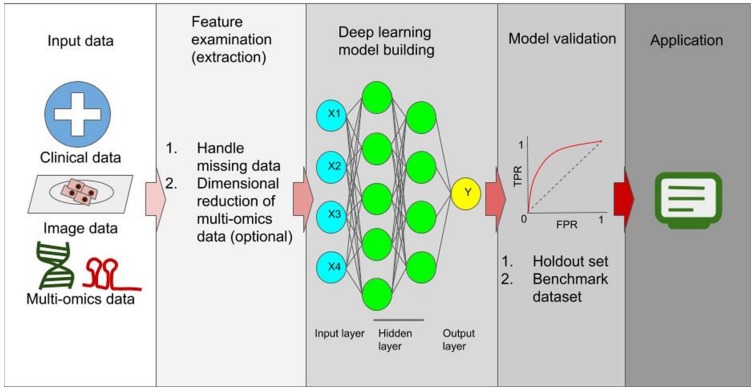
Workflow of building deep learning models for cancer prognosis prediction. The sources of input data include clinical data which could be text data and/or structured data (numeric and/or categorical data), clinical images which could be tissue slides in H&E staining or immune-histological staining. MRI, CT, etc, and genomic data which could be expression data (i.e., mRNA expression data, miRNA expression data), genomic sequence data (i.e., whole genome sequence, SNP data, CNA data, etc), epigenetic data (i.e., methylation data), etc. In the next step, researchers will examine the data to handle missing data and imbalanced data. Reduction of high dimensional genomic data is an optional step here. Features are then used to build a deep learning (neural network) model. The type of models to use depends on the input data. For example, fully connected NN is commonly used for structured datasets. Image data is used to build CNN models. Sequence data is often used to build RNN models. If multiple types of data exist, hybrid models can be built to accept different data types. After the model is built, the model will be tested in the holdout (or validation) datasets. It will also be important to test and compare the models using benchmark datasets. Finally, the model can be used in applications. Abbreviations: FPR: false positive rate; TPR: true positive rate.

By reviewing literature, we have noticed that many state-of-the-art deep learning techniques have been applied to cancer prognosis prediction, indicating the great potential and the urgent need of utilizing multi-omics data from cancer patients to test new algorithm and improve model performance (Figure 1). Meanwhile, we found that there are seven main challenges in applying deep learning approach in cancer prognosis prediction to achieve high performance. We also suggested some potential solutions for these challenges.

First, the amount of patient data is still relatively small. Majority models were built on hundreds of patient samples (Table 1, Table 2 and Table 3). It is common to see sub-optimal performance and overfitting problems in these studies. The performance of deep learning models is leveraged by the amounts of data [27]. To combat overfitting, researchers applied regularization methods (ridge and lasso or L1 and L2), dropout, data augmentation, reduction of NN complexity to improve model performance, but the effect is still limited by the amount of data. To improve model performance with small datasets, transfer learning with pretrain models on large amounts of datasets have shown success solving some of the problems [87,88,89]. In addition, newer methods and algorithms have also been proposed and tested to combat small sample size problem, such as few-shot or one-shot learning in CNN [90,91]. Another direction is to perform data simulation. It will be interesting to test these methods in the field of cancer prognosis.

Second, imbalanced patient data is commonly found. For some high mortality cancers, it is very common to find less survivors in the study groups. Imbalanced data in training will reduce the model performance. While under-sampling in the majority group is suboptimal, generation of synthetic data could be one of the solutions. In image classification problem, data augmentation is also one way to increase sample size to adjust the groups that have fewer sample sizes. Also, reporting model performance should use additional algorithms, such as precision, recall, F1 score and confusion matrix, rather than just reporting accuracy to better reflect the model performance. 

Third, handling sparse or missing data from noisy patient clinical profiles is also a challenge. Missing data in building a model reduce the power of the model in prediction. Common ways to handle missing data include exclusion of missing data observation, but this is very costly when patient samples are already very limited. A better way to overcome this problem is to do data imputation based on known data. Rendleman et al. proposed to perform imputation using Multivariate Imputation by Chained Equations (MICE) [92] to overcome the problem of missing or sparse data in cancer patient outcome [93]. MICE is a multiple imputation technique [94] that works under the assumption that the missing data are missing at random. In this study, they showed that prediction using naive bayes or random forest both works slightly better after imputation, suggesting imputation could be a useful way to improve prediction. 

Fourth, health care data, particularly sequencing data, is high dimensional, feature extraction could be the solution to improve model performance. As we showed in Table 2, studies have performed feature extraction by using algorithm or applying domain knowledge to improve model performance. NN can also be used for feature extraction and dimension reductions [68,86]. It will be interesting to test and apply new way of data embedding for high dimensional data. 

Fifth, more generic deep learning models are needed and model validation in benchmark datasets is critical to validate model performance. The accuracy in model performance is difficult to compare among different studies and different models [95]. Deep learning models with improved algorithms should be built and tested for more generic tasks. For example, a deep recurrent survival analysis which used LSTM cells as the building blocks has been proposed for survival analysis [96]. It will be interesting to test this model in cancer prognosis. Also, building benchmark datasets for model comparison will allow researchers to compare and analyze model performance easier and more efficient. For example, in recent years, ImageNet, a database that contains millions of images from daily life, has been frequently used to evaluate CNN models [97,98,99], which is a critical contributing factor for the development in the field. The models that were built using daily objects from ImageNet have been widely used for other tasks and reach great success. Also, these models are commonly used in many fields and tasks to perform transfer learning. In the medical field, it has also been shown that a single deep learning model is effective at diagnosis across medical modalities [100]. Therefore, building benchmark databases for model validation is urgently needed. One solution is to start building cancer patients’ databases for prognosis analysis [101,102,103,104,105,106,107,108,109,110]. 

Sixth, in addition to technical challenges, building the infrastructure for data storage and establishing the pipeline to build machine learning model may be greatly useful to facilitate the development [8]. Because health care data are sensitive, data safety becomes a concern. How to build a system to safely store and use patients’ health care data to build models and also protect the patients’ privacy requires the effort of administration, research community and personal awareness. Secure cloud services and relevant infrastructure can be established to support the storage of large amount of health care data. Federated learning that only train and predict user data on their own devices is one innovative way to solve privacy issues [111]. 

Lastly, there is the urgent need of researchers who have expertise in biomedical research and machine learning. Compared crowdsourced data annotations, such as annotations for ImageNet objects [112], medical data requires annotators who have expertise to label the data. Domain knowledge facilitate the construction of machine learning models. Therefore, research engineers who have domain knowledge are greatly needed to improve research in this area. To solve this need, universities can provide more relevant courses and degrees. 

## 5. Conclusions and Summary 

Deep learning has made significant improvement in research and started to make changes in our daily lives. In the medical field, many studies have applied deep learning and shown many great successes [78,113,114,115,116,117,118,119,120,121]. One advantage of using deep learning to train a model is its capability to continue training when more data is available [27]. In addition, since health care data have different formats, e.g., genomic data, expression data, clinical (structured) data, text and image (unstructured) data, using different NN architectures to solve different types of data problems become more and more popular and useful [27]. In this review, we summarized recent studies that applied deep learning in studying cancer prognosis (Table 1, Table 2 and Table 3). Among these studies, many have shown deep learning models performed equally or better than other machine learning models [14,58,59]. Future work should continue focusing on testing and improving the algorithm and building state-of-the-art models to improve cancer prognosis prediction.

## 6. Key Points

- Deep learning (DNN) models accept lots of data in different formats. It is a great tool to be used in cancer prognosis prediction since patient’s health data contain multi-source data.
- Using feature extraction could be one way to efficiently extract data from multi-omics data to train neural networks and possibly improve cancer prognosis prediction.
- Fully connected NN and CNN models have been tested in a number of studies to predict cancer prognosis and showed good performance.
- Current deep learning models in cancer prognosis studies still require further testing and validation in larger datasets.

## Figures and Tables

**Table 1:** Summary of neural network models with no feature extraction.

Publication ^a^	Type of Cancer	Type of Data	Sample Size	Methods	Architecture	Outputs	Hyperparameters	Validation	NN Model Performance
Joshi et al., 2006 [58]	Melanoma	Clinical data of tumors	1946 (1160 females and 786 males)	3 layers NN	Normalized input	Survival time	Sigmoid activation	Not reported	Achieved similar performance as Cox and Kaplan Meier statistical methods
Chi et al., 2007 [14]	Breast cancer	Cell images to measure 30 nuclear features	Dataset 1: 198 cases; Dataset 2: 462 cases	3 layers NN	30 input nodes, 20 hidden nods	Survival time	Epoch = 1000, Sigmoid activation	10 fold cross validation	As good as conventional methods
Petalidis et al., 2008 [13]	Astrocytic brain tumor	A list of genes expression from microarray data	65	A single layer perceptron and an output (multiple binary models)	Number of inputs equals to the number of classifier genes in different models	Tumor grades	Lr ^1^ = 0.05, Epoch = 100	Leave-one-out cross validation	44, 9 and 7 probe sets have achieved 93.3%, 84.6%, and 95.6% validation success rates, respectively.
Ching et al., 2018 [59]	10 types of cancer	TCGA gene expression data, clinical data and survival data	5031	NN	Input normalization and log-transformed, 0–2 hidden layers (143 nodes)	Survival time	L1, L2, or MCP ^2^ regularization, tanh activation for hidden layer(s), dropout, Cox regression as output layer	5-fold cross validation	Similar or in some cases better performance than Cox-PH, Cox-boosting or RF
Katzman et al. 2018 [60]	Breast cancer	METABRIC ^3^, 4 genes data and clinical information, GBSG ^4^: clinical data	METABRIC: 1980, GBSG: 1546 training, 686 testing	NN	METABRIC: 1 dense layer, 41 nodes GBSG: patients clinical information, 1 dense layer, 8 nodes	Survival	SELU ^5^ activation, Adam optimizer, dropout, LR decay, momentum	20% of METABRIC patients used as test set GBSG has split test dataset	C-index: 0.654 for METABRIC and 0.676 for GBSG, both are better than CoxPH
Jing et al. 2019 [61]	Breast cancer, nasopharyngeal carcinoma	METABRIC, GBSG, NPC ^7^: 8–9 clinical features	METABRIC: 1980 NPC: 4630	DNN	METABRIC: 4 hidden layers, 45 nodes of each; GBSG: 3 hidden layers, 84, 84 and 70 nodes, respectively NPC, 3 layers, 120 nodes of each layer in model 1, 108, 108 and 90 nodes respectively in model 2.	Survival	ELU ^6^, dropout, L1 and L2, momentum, LR decay, batch size. Loss function equals to mean square error and a pairwise ranking loss	After removed patients with missing data, 20% used as test set	C-index: 0.661 for METABRIC and 0.688 for GBSG, both are better than CoxPH and DeepSurv. c-index ranges 0.681–0.704 depends on input data for NPC, better than CoxPH.

**Abbreviation:**^1^ Lr; learning rate; ^2^ MCP: minimax concave penalty. ^3^ METABRIC: Molecular Taxonomy of Breast Cancer International Consortium; ^4^ GBSG: the German Breast Cancer Study Group; ^5^ SELU: scaled exponential linear unit; ^6^ SELU: exponential linear unit; ^7^ NPC: nasopharyngeal carcinoma. **^a^**Links to source codes if available from publications: Petalidis et al. [13]: http://www.imbb.forth.gr/people/poirazi/software.html. Ching et al. [59]: https://github.com/lanagarmire/cox-nnet. Katzman et al. [60]: https://github.com/jaredleekatzman/DeepSurv. Jing et al. 2019 [61]: http:/github.com/sysucc-ailab/RankDeepSurv.

**Table 2:** Summary of neural network models that used feature extraction.

Publication ^a^	Type of Cancer	Type of Data	Sample Size	Methods Used in Feature Extraction	Architecture	Outputs	Hyperparameters	Validation	NN Model Performance
Sun et al., 2018 [66]	Breast cancer	Gene expression profile, CAN ^1^ profile and clinical data	1980 (1489 LTS ^2^, 491 non-LTS)	mRMR (extracted 400 features from gene expression and 200 features from CNA)	4 hidden layers (1000, 500, 500, and 100 nodes, respectively)	Survival time	Lr ^3^ = 1e–3, Tanh activation, epoch 10–100, batch size = 64	10-fold cross validation	ROC^4^: 0.845 (better than SVM, RF^5^, and LR ^6^), Sp ^7^: 0.794–0.826, Pre ^8^: 0.749–0.875, Sn ^9^: 0.2–0.25, Mcc^10^: 0.356–0.486
Huang et al., 2019 [67]	Breast cancer	mRNA, miRNA, CNB ^11^, TMB ^12^, clinical data	583 (80% for training, 20% for testing in each fold of cross validation)	lmQCM ^13^, Epigengene matrix to extract 57 dimensions from mRNA data and 12 dimensions from miRNA data	Hybrid network, mRNA and miRNA dimension reduction inputs have 1 hidden layer (8 and 4 nodes, respectively), CNB, TMB and clinical data have no hidden layer	Survival time	Adam optimizer, LASSO ^14^ regularization, Epoch = 100, sigmoid activation, Cox regression as output, batch size = 64	5-fold cross validation	Multi-omics data network reached a median c-index^15^ of 0.7285
Hao et al., 2018 [62]	Glioblastoma multiforme	Gene expression (TCGA), pathway (MsigDB ^16^)	475 (376 non-LTS, 99 LTS)	Pathway based analysis (12,024 genes from mRNA data to 574 pathways and 4359 genes)	4 layers NN: gene layer—pathway layer—hidden layer—output	Survival time	Lr = 1e−4, L2 = 3e−4, dropout, softmax output	5-fold validation	AUC^17^ = 0. 66 ± 0.013, F1 score = 0.3978 ± 0.016
Chaudhary et al., 2018 [68]	Liver cancer	mRNA, miRNA, methylation data, and clinical data (TCGA)	360 samples training, (5 additional cohorts, 230, 221, 166, 40 and 27 samples for validation)	Autoencoder unsupervised NN to extract 100 features from mRNA, miRNA and methylation data	3 hidden layers NN (500, 100, 500 nodes, respectively) and a bottleneck layer	Feature reduction	Epoch = 10, Dropout = 0.5, SGD ^18^	Not reported	NN outputs were used for K means clustering.
Shimizu and Nakayama, 2019, [69]	Breast cancer	METABRIC ^19^	1903 (METABRIC, 952 samples for training)	Select 23 genes by statistical methods	3 layers NN	Survival time	Lr = 0.001, Epoch = 1000, Cross entropy for loss function Relu activation for hidden nodes, softmax function for output layer	951 samples from METABRIC	NN node weights were used to calculate a mPS^20^

**Abbreviation: ^1^** CNA: copy number alternation, ^2^ LTS: long term survivals; ^3^ Lr: learning rate; ^4^ ROC: receiver operating characteristic; ^5^ RF: random forest, ^6^ LR: logistic regression, ^7^ Sp: specificity; ^8^ Pre: precision; ^9^ Sn: sensitivity; ^10^ Mcc: Mathew’s correlation coefficient. The equation is (TP*TN-FP*FN)/√ [(TP + FN)*(TP + FP)*(TN + FN)*(TN + FP)]; ^11^ CNB: copy number burden; ^12^ TNB: tumor mutation burden; ^13^ lmQCM: local maximum Quasi Clique Merger [67]; ^14^ LASSO: also known as L1 regularization; ^15^ c-index (concordance index): Steck et al. [70] suggested that c-index is equivalent to AUC. Specifically, c-index closes to 0.5 suggested random prediction. The closer c-index gets to 1, the better the model is. ^16^ MsigDB: Molecular Signatures Database; ^17^ AUC: area under the curve of ROC; ^18^ SGD: stochastic gradient descent; ^19^ METABRIC: Molecular Taxonomy of Breast Cancer International Consortium; ^20^ mPS: molecular prognostic score. **^a^** Links to source codes if available from publications: Sun et al., 2018 [66]: https://github.com/USTC-HIlab/MDNNMD. Huang et al., 2019 [67]: https://github.com/huangzhii/SALMON/. Hao et al., 2018 [62]: https://github.com/DataX-JieHao/PASNet. Shimizu and Nakayama, 2019, [69]: https://hideyukishimizu.github.io/mPS_breast.

**Table 3:** Summary of CNN based models.

Publication ^a^	Type of Cancer	Type of Data	Sample Size	Architecture	Outputs	Hyperparameters	Validation	NN Model Performance
Korfiatis et al., 2017 [81]	Glioblastoma multiforme	MRI images	155 (66 methylated and 89 unmethylated tumors) Training: 7856 images (934 methylated, 1621 unmethylated, 5301 no tumor) Testing: 2612 images (250 methylated, 335 unmethylated, 2027 no tumor)	Base model: ResNet18 ResNet34 ResNet50	3 classes, methylated, unmethylated, or no tumor	Lr^1^ = 0.01, mini Batch = 32, momentum = 0.5, weight decay = 0.1, Relu activation, Epoch = 50, SGD^2^ as optimizer, batch normalization	Stratified cross-validation	ResNet50 based model validation dataset performance: Accuracy = 94.9%, Precision = 96%, Recall = 95% ResNet34 Accuracy = 80.72%, Precision = 93%, Recall = 81%, ResNet18 Accuracy = 76.75%, Precision:80%, Recall = 77%
Han et al., 2018 [81]	Glioblastoma multiforme	MRI images	458,951 image frames from 5235 MRI scans of 262 patients (TCIA^3^)	3 convolutional layers, 2 fully connected layers, 1 bi-directional GRU^4^ layer (RNN), 1 fully connected layer, softmax output	2 classes (positive and negative methylation status)	Data augmentation, (rotation and flipping, 90-fold increase of the dataset), Lr = 5e−6 – 5e−1, dropout (0–0.5), Adam optimizer, Epoch = 10, L2 regularization, batch norm, relu activation	Validation set reached a precision of 0.67, an AUC of 0.56.	Training data set obtained 0.97 accuracy. 0.67 and 0.62 accuracies on the validation and test set, respectively
Mobadersany et al., 2018 [83]	Low grade glioma and glioblastoma	H&E images, genomics data, clinical data	769 gliomas from TCGA, containing genomics data (IDH mutation and 1 p/19 q codeletion), clinical data and 1061 slides.	VGG19 is the base model and cox regression used as output, Built 2 models with or without genomics data	Survival	Data augmentation, Lr = 0.001, epoch = 100, exponential learning decay	Monte Carlo cross-validation	SCNN median c-index is 0.754, GSCNN (adding IDH mutation and 1 p/19 q codeletion as features) improved the median c-index to 0.801
Kather et al., 2019 [74]	Colorectal cancer	H&E tissue slides	Training set (tissue): 86 H&E slides to create 100,000 image patches Testing set (tissue): 25 H&E slides of 7180 image patches Training set (OS^5^): 862 H&E slides from 500 TCGA patients Validation set (OS): 409 H&E slides from 409 DACHS patients	Base models: VGG19, AlexNet, GoogLeNet, SqueezeNet and Resnet50, add an output softmax layer	9 tissue type classification	Lr = 3e −4, Iteration = 8, Batch size = 360, softmax function	An independent cohort of 409 samples	VGG19 gets the best results, 94–99% accuracy in tissue class prediction
Bychkov et al., 2018 [84]	Colorectal cancer	H&E images of tumor tissue microarray	420 patients (equal number of survived or died within five years after diagnosis)	VGG16 to generate a 16 × 16 feature from input data, followed with 3 layers LSTM ^6^ (264, 128 and 64 LSTM cells, respectively)	Survival	Default hyperparameters in VGG16, LSTM used hyperbolic tangent as activation, binary cross entropy loss function, Adadelta optimizer	60 samples for validation, 140 samples for testing	CNN + LSTM model reached an AUC ^7^ of 0.69, better than CNN + SVM, CNN + LR ^8^, or CNN + NB ^9^
Courtiol et al., 2019 [85]	Mesothelioma	H&E slides	2981 patient slides (MESOPATH/MESOBANK, 2300 training, 681 testing) Validation: 56 patients (TCGA)	Divided each slide to up to 10,000 tiles as input data 3 classes of each tile: epithelioid, sarcomatoid or biphasic. ResNet50 for feature extraction	Survival	Multi-layer perceptron with sigmoid activation, Autoencoder	56 patient slides	MesoNet outperformed histology-based classification but no better than a linear regression based model (Meanpool)
Wang et al., 2019 [86]	High grade serous ovarian cancer	CT scanning venous phase images	Feature learning cohort: 8917 CT images from 102 patients	Five convolutional layers (24, 16, 16, 16, 16 filters, respectively)	16 dimensional feature vector	Batch normalization, average pooling between adjacent convolutional layers	Not reported	CNN outputs were used to build Cox-PH model

Abbreviation: ^1^ Lr: learning rate; ^2^ SGD; stochastic gradient descent; ^3^ TCIA: The Cancer Image Archive; ^4^ GRU: gated recurrent unit, which is similar to LSTM and is used in building RNN models; ^5^ OS: overall survival; ^6^ LSTM: long short term memory cell; ^7^ AUC: area under the curve of ROC; ^8^ LR: logistic regression; ^9^ NB: naive bayes; ^10^ c-index: also known as Harell’s concordance index. **^a^** Links to source codes if available from publications: Han et al., 2018 [81]: http://onto-apps.stanford.edu/m3crnn/. Kather et al., 2019 [74]: http://dx.doi.org/10.5281/zenodo.1214456,http://dx.doi.org/10.5281/zenodo.1420524, http://dx.doi.org/10.5281/zenodo.1471616, Wang et al., 2019 [86]: http://www.radiomics.net.cn/post/111.
